# MOTS‐c increases in skeletal muscle following long‐term physical activity and improves acute exercise performance after a single dose

**DOI:** 10.14814/phy2.15377

**Published:** 2022-07-08

**Authors:** Jon‐Philippe K. Hyatt

**Affiliations:** ^1^ College of Integrative Sciences and Arts Arizona State University Tempe Arizona USA

**Keywords:** detraining, medial gastrocnemius, mouse, plantaris, rat, tibialis anterior

## Abstract

Skeletal muscle adapts to aerobic exercise training, in part, through fast‐to‐slow phenotypic shifts and an expansion of mitochondrial networks. Recent research suggests that the local and systemic benefits of exercise training also may be modulated by the mitochondrial‐derived peptide, MOTS‐c. Using a combination of acute and chronic exercise challenges, the goal of the present study was to characterize the interrelationship between MOTS‐c and exercise. Compared to sedentary controls, 4–8 weeks of voluntary running increased MOTS‐c protein expression ~1.5‐5‐fold in rodent plantaris, medial gastrocnemius, and tibialis anterior muscles and is sustained for 4–6 weeks of detraining. This MOTS‐c increase coincides with elevations in mtDNA reflecting an expansion of the mitochondrial genome to aerobic training. In a second experiment, a single dose (15 mg/kg) of MOTS‐c administered to untrained mice improved total running time (12% increase) and distance (15% increase) during an acute exercise test. In a final experiment, MOTS‐c protein translocated from the cytoplasm into the nucleus in two of six mouse soleus muscles 1 h following a 90‐min downhill running challenge; no nuclear translocation was observed in the plantaris muscles from the same animals. These findings indicate that MOTS‐c protein accumulates within trained skeletal muscle likely through a concomitant increase in mtDNA. Furthermore, these data suggest that the systemic benefits of exercise are, in part, mediated by an expansion of the skeletal muscle‐derived MOTS‐c protein pool. The benefits of training may persist into a period of inactivity (e.g., detraining) resulting from a sustained increase in intramuscular MOTS‐c proteins levels.

## Graphical abstract

Exercise performance is enhanced 12‐15% in adult female mice following an acute supplementation of the MOTS‐c peptide.

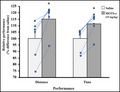

## 1 INTRODUCTION

Skeletal muscle adapts to long‐term aerobic exercise, in part, by expanding mitochondrial volume (Meinild Lundby et al., 2018), augmenting capillary density (Klausen et al., 1981), and undergoing a fast‐to‐slow phenotypic shift (Fuller et al., 2006). These tissue‐specific changes contribute to an overall improvement in glucose maintenance, cardiovascular dynamics, and general fitness as reflected in total oxygen consumption (e.g., VO_2_), which, collectively, enhances an individual's competitive performance (Holloszy & Coyle, 1984).

Mitochondrial open reading frame (ORF) of the 12S rRNA type‐c (MOTS‐c) has emerged as a strong candidate for influencing intracellular communication and whole‐body physiology (Kim et al., 2017; Kim et al., 2018; Lee et al., 2016; Li & Laher, 2015, Lu, Tang, et al., 2019; Reynolds et al., 2021; Yuan et al., 2021). MOTS‐c is abundantly expressed in skeletal muscle (D'Souza et al., 2020; Lee et al., 2015) and functions, in part, to increase glucose metabolism by enhancing insulin sensitivity, energy expenditure, and heat production (Lee et al., 2015). Circulating MOTS‐c decreases with fasting (Lee et al., 2015) and increases during short, high‐intensity exercise (Reynolds et al., 2021; von Walden et al., 2021). In cultured conditions of metabolic stress (i.e., glucose restriction/serum deprivation, oxidative stress), MOTS‐c translocates into the nucleus to influence gene expression in an AMPK‐dependent manner (Kim et al., 2018). Although the potential benefits of MOTS‐c supplementation have been emphasized in aged/pathophysiological paradigms (Kim et al., 2019; Lee et al., 2015; Lu, Wei, et al., 2019), recent work indicates that 2 weeks of daily MOTS‐c preconditioning also improves exercise performance in young healthy adult mice (Reynolds et al., 2021).

MOTS‐c protein expression is greater in slow than in fast skeletal muscle (D'Souza et al., 2020), but it is unknown whether MOTS‐c levels parallel the adaptations that occur in skeletal muscle in response to exercise training or detraining. Chronic aerobic exercise training, for example, induces a fast‐to‐slow phenotypic shift (Allen et al., 2001; Demirel et al., 1999; Fuller et al., 2006; Hyatt et al., 2019; Moreillon et al., 2019; Short et al., 2005; Tajsharghi et al., 2004) which is accompanied by a concomitant increase in mitochondrial DNA (mtDNA) content (Constantin‐Teodosiu et al., 2020; Crane et al., 2013; Lee et al., 2018), likely reflecting an expansion in skeletal muscle mitochondrial volume (Meinild Lundby et al., 2018). These exercise‐induced adaptations eventually return to basal levels if training ceases (e.g., detraining), although the time course of this return is dependent on a number of conditions including, but not limited to, the peak level of fitness achieved with training and the length of detraining (Hyatt et al., 2019). The goal of this study was, in part, to test whether MOTS‐c protein expression similarly adapted to chronic exercise training. It was hypothesized that with an expansion in mtDNA in response to chronic exercise training, MOTS‐c protein expression would similarly increase in trained skeletal muscles but return to basal levels following a period of detraining. Secondly, considering previous work showed that intramuscular MOTS‐c protein accumulates with daily MOTS‐c injections (Lee et al., 2015) and that several weeks of MOTS‐c preconditioning was required for performance enhancement (Reynolds et al., 2021), it was hypothesized that a single dose of MOTS‐c administered to young adult mice would have no effect on performance with an acute exercise challenge. In a final experiment to test MOTS‐c translocation in vivo as previously demonstrated in vitro (Kim et al., 2018), it was hypothesized that MOTS‐c would translocate to skeletal muscle nuclei from cytoplasmic and/or mitochondrial regions when challenged with an acute downhill running test.

## 2 METHODS

### 2.1 Animals and physical activity protocols

All procedures and treatment protocols for animals used onsite were approved by the Arizona State University Institutional Animal Care and Use Committee in accordance with the guidelines of the American Physiological Society. All rodents were given a minimum of 48 h to acclimatize to the new housing environment upon arrival and allowed ad libitum access to standard rodent chow and water.

### 2.2 Chronic physical activity experiments

It should be noted that all timepoints of chronic physical activity using rats were conducted separately and completed before the MOTS‐c hypotheses were conceptualized; all rat muscles were frozen before MOTS‐c analyses began and only the soleus and plantaris muscles were dissected and stored from the rats of these groups. Early analysis of the rat plantaris samples suggested a positive association between MOTS‐c protein expression and chronic physical activity. To determine whether this initial observation in rats generalized to another rodent species and to hindlimb muscles that are functionally and phenotypically different than the rat plantaris (Allen et al., 2001), a cohort of mice were added to the chronic physical activity experiments that fell within the 4–8 week time frame of trained rats.

Young adult female Sprague–Dawley rats (~120 g) (Envigo) were randomly assigned to groups upon arrival. A sedentary (SED; *n* = 11) control group was housed as pairs in standard cages for 8 weeks. An exercise‐trained group consisted of rats that were housed individually in cages with voluntary access to resistance‐free running wheels (#80859S; Lafayette Instrument) for 6 weeks (EX6; *n* = 6). A second group of rats voluntarily trained for 6 weeks followed by a sedentary period (e.g., detraining) that involved housing in standard cages for an additional 6 weeks (DETR6; *n* = 6). Daily running distances were recorded by Scurry software (Lafayette) and running wheels were calibrated weekly. The rats from these protocols supplemented timepoints from a previous study (Hyatt et al., 2019) that included: 4 weeks of voluntary exercise (EX4; *n* = 10), 8 weeks of voluntary exercise (EX8; *n* = 8), and 4 weeks of exercise followed by 4 weeks of detraining (DETR4; *n* = 9). The rats and equipment used during this and our early study (Hyatt et al., 2019) were identical.

**FIGURE 1:**
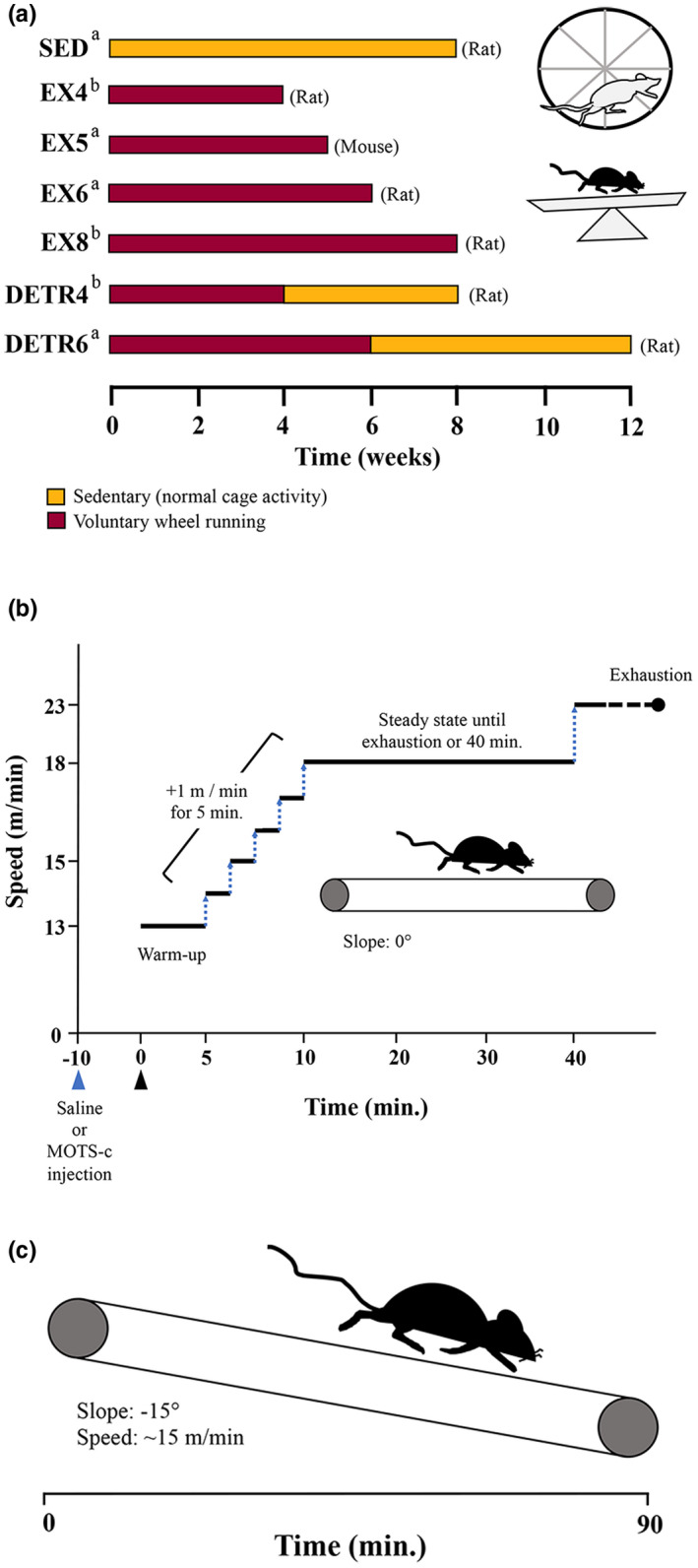
Schematic overview of the experimental designs employed for chronic physical activity (a), an acute exercise test following MOTS‐c supplementation (b), and an acute downhill running challenge (c). (a) Changes in MOTS‐c protein expression were examined in muscles of sedentary (SED), exercise‐trained (EX), or detrained (DETR) adult female Sprague Dawley rats or C57BL/6j mice given access to resistance‐free up‐right running wheels (rats) or low‐profile wheels (mice) for 4 (EX4; *n* = 10), 5, (EX5; *n* = 6), 6 (EX6; *n* = 6), or 8 (EX8; *n* = 8) weeks. DETR4 (*n* = 9) rats voluntarily trained for 4 weeks followed by 4 weeks of sedentary activity, whereas DETR6 (*n* = 6) rats voluntarily trained for 6 weeks followed by 6 weeks of sedentary activity. Rodents groups in the present study (a) were combined with groups from earlier work (b; Hyatt et al., 2019) for comparisons. (b): To examine the effect of a single MOTS‐c supplement on exercise performance, adult female C57BL/6j mice (*n* = 6) were injected (blue triangle) with saline or MOTS‐c peptide (15 mg/kg) 10 min prior to the start of an acute exercise test (black triangle), which consisted of a warm up (5 min at 13 m/min), an incremental increase in speed (+1 m/min for 5 min) until steady‐state was reached (18 m/min). Mice ran at steady‐state until exhaustion or 40 min total run time, at which time the speed was increased to 23 m/min until exhaustion was reached. (c) A downhill running challenge tested whether MOTS‐c protein translocated to skeletal muscle nuclei immediately, 1 h, and 1 day post‐exercise (*n* = 6/group). The treadmill was set at −15° and mice ran at ~15 m/min for 90 min.

Young adult female C57BL/6j (10 weeks old) (Jackson Labs) were randomly assigned to groups upon arrival. A sedentary (SED; *n* = 6) control group of mice were housed in groups of three in standard cages for 5 weeks. An exercise‐trained group of mice (EX5; *n* = 6) were placed individually in cages for 5 weeks with voluntary access to resistance‐free running low‐profile wheels (#86180; Lafayette), which are preferred by the mice compared to up‐right/vertical wheels (Manzanares et al., 2018). Allen et al. (2001) observed that most skeletal muscle adaptations to voluntary wheel running occurred after 4 weeks; here, a 5‐weeks timepoint was selected to ensure sufficient aerobic adaptation had occurred. Daily mouse running distances were recorded by ClockLab data collection software and the wheels were calibrated weekly. Despite the differences in time points (4, 5, 6, or 8 weeks) and rodent species (rats or mice), the central question in this study was the same: Does MOTS‐c expression change in skeletal muscles following chronic physical activity? A summary of rodent groups and conditions are presented in Figure 1a.

### 2.3 Acute physical activity experiments

C57BL/6j mice (11 weeks old; *n* = 6) were challenged with an acute exercise test after receiving either a saline or synthetic MOTS‐c peptide supplement. Using a cross‐over design, all mice experienced this acute exercise test twice: Once after receiving a 0.9% saline supplement and once after receiving a MOTS‐c supplement. For the first bout of exercise, three mice received saline and the remaining mice were supplemented with MOTS‐c; for the second bout, the supplement was switched for each mouse. Exercise bouts were separated by 10 days. Ten minutes before the exercise challenge started, 110 μl of saline or full‐length (Lee et al., 2015) human MOTS‐c peptide (15 mg/kg; Genscript) was injected intraperitoneally. The exercise test, which was performed on a level treadmill, was identical to that previously reported (Reynolds et al., 2021). Briefly, the mice warmed up for 5‐min at 13 m/min. Over the next 5 min, the speed was increased by 1 m/min. The goal was for the mice to run at 18 m/min for 30 min until a total run time of 40 min was obtained. However, if a mouse demonstrated signs of exhaustion, which was identified as a mouse sitting on the shock grid for ~5 s (Poole et al., 2020), then the exercise test was terminated for that animal and the time was recorded. For all mice that reached the 40‐min timepoint, the running speed was increased to 23 m/min until exhaustion was achieved and the time was recorded. A summary of the acute exercise test protocol is shown in Figure 1b.

A final cohort of mice was challenged with a downhill running exercise to determine whether MOTS‐c translocated to nuclei in vivo as previously observed under cell culture conditions of metabolic stress (Kim et al., 2018). Downhill running in rodents was chosen as the exercise paradigm because it induces mechanical (eccentric contractions) and metabolic stress on working skeletal muscles as reflected in glycogen depletion (Ferry et al., 1992; Hesselink et al., 1998) oxidative stress/mitochondrial dysfunction (Hody et al., 2022; Magalhães et al., 2013), and the presence of central myonuclei/myonuclear accretion (Hesselink et al., 1998; Luis Araujo Minari et al., 2022). Young adult female C57BL/6j (11 weeks old) were randomly assigned to one of four groups (*n* = 6 per group): Non‐exercise control (CON), immediately post‐ (Imm.), 1 h post‐, and 1d post‐exercise. Mice were exercised individually on a treadmill (Columbus Instruments) at −15° for 90 min (Figure 1c). During this challenge, the objective was to maintain a speed ≥15 m/min for all mice; however, because the mice were untrained, all animals were carefully monitored for signs of fatigue including trailing toward the back of the treadmill and repeated touches on the shock grid. If these indicators were observed, which occurred with increased frequency toward the end of the 90‐min period, then the treadmill speeds were reduced to allow the mice to finish the 90‐min test. All mice in all groups completed the downhill running exercise averaging (mean ± SD) 15.57 ± 1.59 m/min equating to 1401 ± 143 m in total distance. All mice were euthanized within 2 min of the pre‐determined group time points (i.e., Imm.‐, 1 h‐, or 1 d‐post exercise).

At the end of each experiment, the animals were euthanized with an overdose of carbon dioxide. The plantaris muscles from each rat were removed bilaterally, trimmed of excess connective tissue, wet weighed, pinned to cork at the approximate in situ resting length, and frozen in isopentane cooled by liquid nitrogen. For 5‐week exercise‐trained mice, the medial gastrocnemius (MG) and tibialis anterior (TA) were removed bilaterally and frozen as described above. All frozen samples were stored at −80°C until further analysis. For the mice challenged with downhill running, the soleus and plantaris muscles were removed bilaterally, wet‐weighed, and placed in ice‐cold saline prior to homogenization and separation into nuclear, cytoplasmic, and mitochondrial fractions (see below).

### 2.4 mtDNA abundance

Total DNA was extracted using a modified protocol detailed by Strauss (1998). A 15–30 mg chunk was removed from the mid‐belly of rat or mouse skeletal muscles and digested at 40°C for 18 h in buffer containing 1 M NaCl, 0.1 M Tris–HCl, 0.1 M EDTA, 10% SDS, and fresh 0.1 mg/ml proteinase K. DNA was then extracted using standard phenol:chloroform:isoamyl alcohol methods (Strauss, 1998) and resuspended in 1X Tris‐EDTA buffer (pH 8.0). Semi‐quantitative end‐point PCR was performed using primers generated against nuclear (nDNA) targets for rat (actin beta; *ACTB*) and mouse (β2 microglobulin; *B2M*). Primer pairs for mitochondrial‐encoded rat or mouse NADH Dehydrogenase 1 (*ND1*) were generated and second primer pair was developed against rat ATP synthase subunit 6 (*ATP6*) and mouse cytochrome c oxidase subunit I (*COX1*). Primer sequences for each are shown in Table 1. Samples were prepared using 2X master mix containing optimized concentrations of *Taq* polymerase, dNTPs, MgCl_2_ (Promega); 30 ng of DNA per reaction per sample was used. A cycles test spanning 10–34 cycles was performed to determine the optimal number of PCR cycles for each gene (Table 1). The cycling conditions consisted of one cycle at 95°C for 2 min followed by 95°C for 30 s, 58.5°C for 45 s, and 72°C for 45 s for the pre‐determined cycle number for each amplicon (Table 1). A final extension at 72°C for 5 min competed the amplification (Benchmark). Amplicons were separated in a 0.8% agarose gel containing 1X GelGreen (Biotium) at 45 V for 60 min, photographed and quantified using ImageJ software (Rasband, 1997–2018). The mitochondrial‐to‐nuclear gene ratio was calculated for each sample, averaged for each group, and statistically compared.

**TABLE 1:** Primer sequences used for PCR analysis

Target	Species	Primer sequences (5′→3′)		Cycles	Size (bp)
*ACTB*, Actin Beta	Rat	AGCTGAGAGGGAAATCGTGC	Forward	27	501
		ACACCCCACTATGGGTCCAG	Reverse		
β2 microglobulin	Mouse	CTGGGGTAAGCCTCAAGTTC	Forward	27	296
		GGCAGGGGTTACAGACCAAG	Reverse		
*ND1*, NADH Dehydrogenase 1	Rat	TAAGCGGCTCCTTCTCCCTA	Forward	16	368
		GGGGTAGGATGCTCGGATTC	Reverse		
	Mouse	CTAGCAGAAACAAACCGGGC	Forward	20	284
		TGATCGTAACGGAAGCGTGG	Reverse		
*ATP6*, ATP synthase subunit 6	Rat	AACGCCTAATCAGCAACCGA	Forward	16	227
		TGCTCATAGGGGGATGGCTA	Reverse		
*COX1*, cytochrome c oxidase subunit I	Mouse	GAGCGGGAATAGTGGGTACTG	Forward	20	349
		GCTCCTGCATGGGCTAGATT	Reverse		

### 2.5 Protein isolation

Muscle chunks for total protein isolates were acquired from the ipsilateral side that was used for PCR analysis. Because the mid‐belly region was used for total DNA isolation the chunks for protein isolation were cut proximally from the original mid‐belly cut. Approximately 15–30 mg of muscle was used for homogenization. Samples were bead‐ homogenized for 2.5 min at 3000 rpm in 10‐volume ice‐cold buffer (pH 7.8) containing 50 mM Tris–HCl, 2 mM EDTA, 2 mM EGTA, 10% glycerol, 1% Triton‐X, and a 1% v/v final concentration of a protease and phosphatase inhibitor cocktail (PPC1010; Sigma) containing 4‐(2‐Aminoethyl) benzenesulfonyl fluoride hydrochloride, aprotinin, bestatin hydrochloride, *N*‐(trans‐Epoxysuccinyl)‐l‐leucine 4‐guanidinobutylamide, leupeptin, pepstatin A, cantharidin, (−)‐p‐Bromolevamisole oxalate, and calyculin A. After homogenization, the samples were centrifuged at 12,000 *g* for 10 min at 4°C and the supernatant was transferred to clean tubes and frozen at −80°C.

Following the downhill running experiments, protein was isolated into cellular fractions from whole mouse soleus and plantaris muscles using a modified protocol by Frezza et al. (2007). Briefly, fresh muscle was coarsely minced with a razor blade, transferred to ice‐cold in 10‐volume IBm_1_ buffer and homogenized using a glass/teflon Potter Elvehjem homogenizer. The sample was centrifuged at 700 *g* for 10 min at 4°C; the pellet was collected as the nuclear fraction, placed in clean a tube, and washed 3× in ice‐cold 750 μl IBm_1_ buffer to remove any residual cytoplasmic or mitochondrial protein; between washes, the nuclear fraction was pelleted 700 *g* for 10 min at 4°C. The nuclear fraction was finally resuspended in 4.5‐volume of IBm_1_ buffer, freeze‐thawed 3×, and passed through a 25G syringe to sheer the genomic DNA. The supernatant remaining from the original nuclear pellet fraction was centrifuged at 8000 *g* for 10 min at 4°C. The subsequent supernatant was collected as the cytosolic fraction and placed in clean tubes. The remaining pellet (mitochondrial fraction) was washed 3× in ice‐cold IBm_2_ buffer. Between washes, the mitochondrial pellet was centrifuged at 8000 *g* for 10 min at 4°C. Finally, the pellet of the mitochondrial fraction was resuspended in a 12–20 μl of IBm_2_ buffer, freeze‐thawed 3×, and stored at −80°C. The concentration of total, nuclear, cytoplasmic, and mitochondrial protein was determined using the Bio‐Rad Protein Assay (Bio‐Rad) from a small aliquot of isolate; the remainder of each sample was stored at −80°C until Western analysis.

### 2.6 Western analysis

Loading of total protein for immunoblotting ranged between 10 and 50 μg depending on the target (Hyatt et al., 2022). Total protein was separated using either 10%, 12%, or 15% acrylamide tris‐glycine gels. Samples were mixed in 2× sample buffer (0.2% SDS, 20% glycerol, 25% 4× buffer, 5% β‐mercaptoethanol, and 0.025% bromophenol blue) and heated to either 42.5°C when probing membranes for COX1 or 100°C for 3 min and electrophoresed at 80 V for 20 min and then 140 V for 60–70 min. The proteins were wet‐transferred to PVDF membranes for 100 min at 50 V for any target <20 kDa or for 3 hr at 60 V. The membranes were then placed in a solution of Ponceau S (Sigma) to verify that the transfer was uniform and artifact‐free, partially de‐stained in ddH_2_O, and digitally scanned for sample loading control used in subsequent analyses and quantification. The membranes were then placed in 5% nonfat dry milk (NFM) dissolved in tris‐buffered saline with 0.05% Tween‐20 (T‐TBS) for a minimum of 0.5 h. The membranes were incubated in primary antibody diluted in NFM for 1 h at room temperature or overnight at 4°C. The primary antibodies and dilutions used were: COX1 (OXPHOS cocktail, Abcam Inc.; ab110413; 1:1000), Cytochrome oxidase B (CYTB, Proteintech; 55090‐1‐AP; 1:1000); ATP6 (Proteintech, 55313‐1‐AP; 1:1000), and Humanin (Sigma, H2414; 1:1000).

**FIGURE 2:**
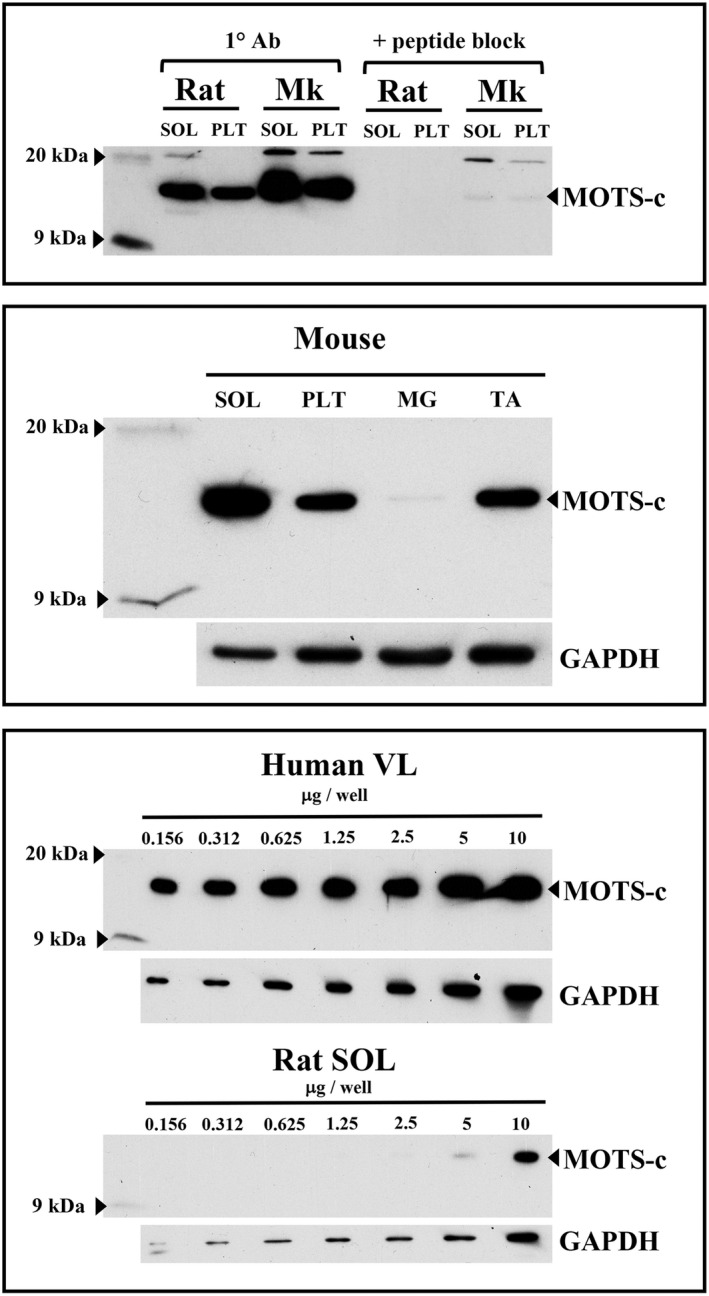
MOTS‐c antibody (1° Ab) an reactivity and specificity in control rat, mouse, rhesus macaque (Mk), and human skeletal muscle. MOTS‐c was identified at ~14 kDa following a 1‐h peptide block against a rat MOTS‐c peptide (top) and was expressed highest in the phenotypically slow mouse soleus (SOL) and almost undetectable in the fast mouse medial gastrocnemius (MG) muscle (middle). Serially diluted samples show specificity of the MOTS‐c antibody in human vastus lateralis (VL) and rodent (rat) SOL muscles (bottom). GAPDH protein was used as a loading control. PLT, plantaris; TA, tibialis anterior.

A custom anti‐rat rabbit polyclonal antibody was developed (Abcam, 1:5000) against MOTS‐c using the source sequence, from N‐ to C‐terminus, MKRKEMGYIFF (Lee et al., 2015). Detection of the MOTS‐c protein was tested in control rat, mouse, rhesus macaque, and human skeletal muscle samples. Human vastus lateralis samples were generously provided by C. Katsanos (Arizona State University). To confirm specificity, ~1 μg of antibody was spiked with 3 μg of rat MOTS‐c peptide (GenScript) for 1 h at room temperature prior to incubation with a membrane containing samples. Skeletal muscle MOTS‐c was detected at ~14 kDa (Figure 2). After primary antibody incubation, membranes were washed 6 × 10 min in T‐TBS and incubated for 1 h in a secondary antibody dilution for 1 h at room temperature. The membranes were developed using an ECL detection kit (GE Healthcare Bio‐Sciences Corp.) per the manufacturer's instructions. Densitometry and quantification were performed using ImageJ software (Rasband, 1997–2018).

### 2.7 Statistics

Values are presented as means ± SE. A one‐way analysis of variance (ANOVA) was performed to ascertain overall differences between groups for all variables and a Student's one‐tailed test using a Bonferroni correction adjustment was performed for post hoc comparisons. For all statistical analyses, SPSS (v24) was used and significance was set at *p* < 0.05.

## 3 RESULTS

### 3.1 Running distances and muscle masses following chronic physical activity

Trained rats ran voluntarily for either 4 (EX4), 6 (EX6), or 8 weeks (EX8) using resistance‐free running wheels. In parallel, DETR4 and DETR6 rats voluntarily trained for 4 or 6 weeks and then detrained (normal cage activity) for 4 or 6 weeks, respectively. Total running distance (Table 2) for each group of rats depended on the total time in the presence of the running wheels. EX4 rats ran a comparable distance (~550 km) that the DETR6 and EX8 rats ran in 6 (~520 km) and 8 weeks (560 km), respectively. Conversely, the total running distance DETR4 rats were significantly less than EX4 and EX8 rats (*p* < 0.05). Similarly, absolute and relative plantaris muscle masses (Table 3) reflected the relative age with which group experiments concluded: EX4 rats were euthanized at a younger at age than other groups and the absolute muscle mass was, therefore, significantly lighter (*p* < 0.05). EX8 and DETR4 relative muscle masses also were significantly heavier than EX4 rats. No differences in SED and EX5 mouse MG and TA absolute and relative muscle masses were detected (Table 3).

**TABLE 2:** Total and daily running distances (mean ± SE) for rat and mouse sedentary (SED), exercise (EX) and detraining (DETR) groups

Group	*n*	Total distance (km)	Daily distance (km)
Rat			
SED	11	0	0
EX4	10	551.6 ± 61.0^a^	15.8 ± 1.8^b^
EX6	6	371.3 ± 94.7	8.9 ± 2.3
EX8	8	560.0 ± 71.2^a^	8.8 ± 1.1
DETR4	9	321.9 ± 44.5	9.8 ± 1.4
DETR6	6	519.3 ± 27.5	11.5 ± 0.6
Mouse			
EX5	6	536.5 ± 52.8	15.3 ± 1.5

^a^ Different from DETR4 (*p* < 0.05).

^b^ Different from EX6, EX8, and DETR4 daily running distances (*p* < 0.05).

**TABLE 3:** Absolute (mg) and relative (mg/g) muscle masses for

	Absolute mass (mg)	Relative mass (mg/g)
Rat		
PLT		
SED	295 ± 9.6	1.18 ± 0.02
EX4	210.3 ± 4.6^a^	1.11 ± 0.02
EX6	259.5 ± 9.5	1.12 ± 0.03
EX8	273.5 ± 13.9	1.21 ± 0.02^c^
DETR4	301.1 ± 7.3^b^	1.23 ± 0.03^c^
DETR6	317.8 ± 12.0^b^	1.23 ± 0.05
Mouse		
MG		
SED	60.67 ± 1.91	2.87 ± 0.10
EX5	60.33 ± 1.74	2.94 ± 0.11
TA		
SED	43.0 ± 1.47	2.03 ± 0.07
EX5	40.2 ± 0.96	1.96 ± 0.02

^a^ Different from all groups (*p* < 0.05).

^b^ Different from EX6 (*p* < 0.05).

^c^ Different from EX4 (*p* < 0.05).

### 3.2 Chronic physical activity enhances mtDNA content and MOTS‐c protein expression in rodent skeletal muscle

**FIGURE 3:**
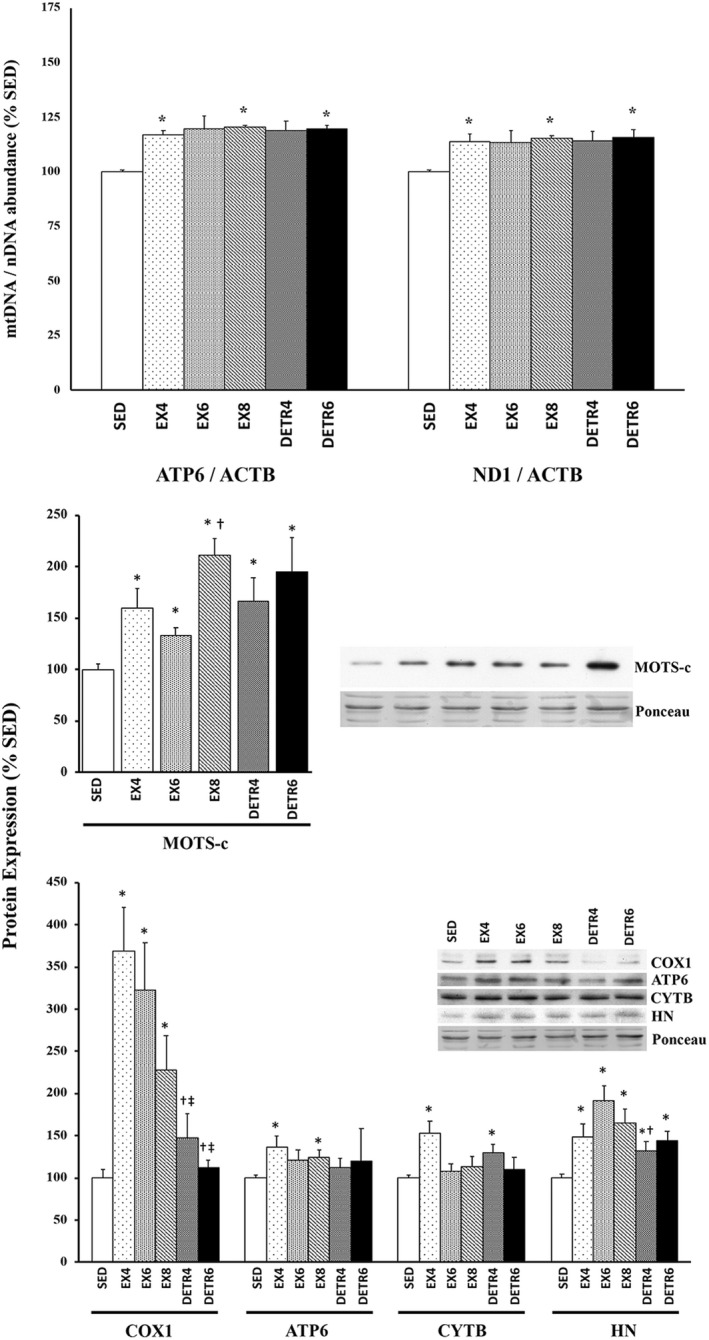
Changes in mitochondrial DNA (mtDNA) abundance (top), MOTS‐c protein (middle), and mitochondrial‐encoded proteins (bottom) in female Sprague–Dawley rat plantaris muscle after long‐term voluntary aerobic training. Group comparisons were made between sedentary (SED) and 4‐ (EX4; *n* = 10), 6‐ (EX6; *n* = 6), and 8‐week (EX8; n = 8)‐trained rats as well as detrained rats that were trained for 4 or 6 weeks followed by 4 (DETR4; n = 9) or 6 (DETR6; *n* = 6) weeks of detraining, respectively. The mtDNA‐to‐nuclear DNA (nDNA) ratios determined using mitochondrial genes ATP synthase subunit 6 (ATP6) and NADH Dehydrogenase 1 (ND1) and the nuclear housekeeping gene, actin beta (ACTB). Expression of cytochrome c oxidase subunit I (COX1), ATP6, cytochrome b (CYTB), and humanin (HN) proteins are shown; representative protein and ponceau‐stained membranes as a loading control are shown. Values show mean ± S.E. *Statistically different from SED (*p* < 0.05); ^†^Sig from EX6 (*p* < 0.05); ^‡^Sig from EX4 (*p* < 0.05).

**FIGURE 4:**
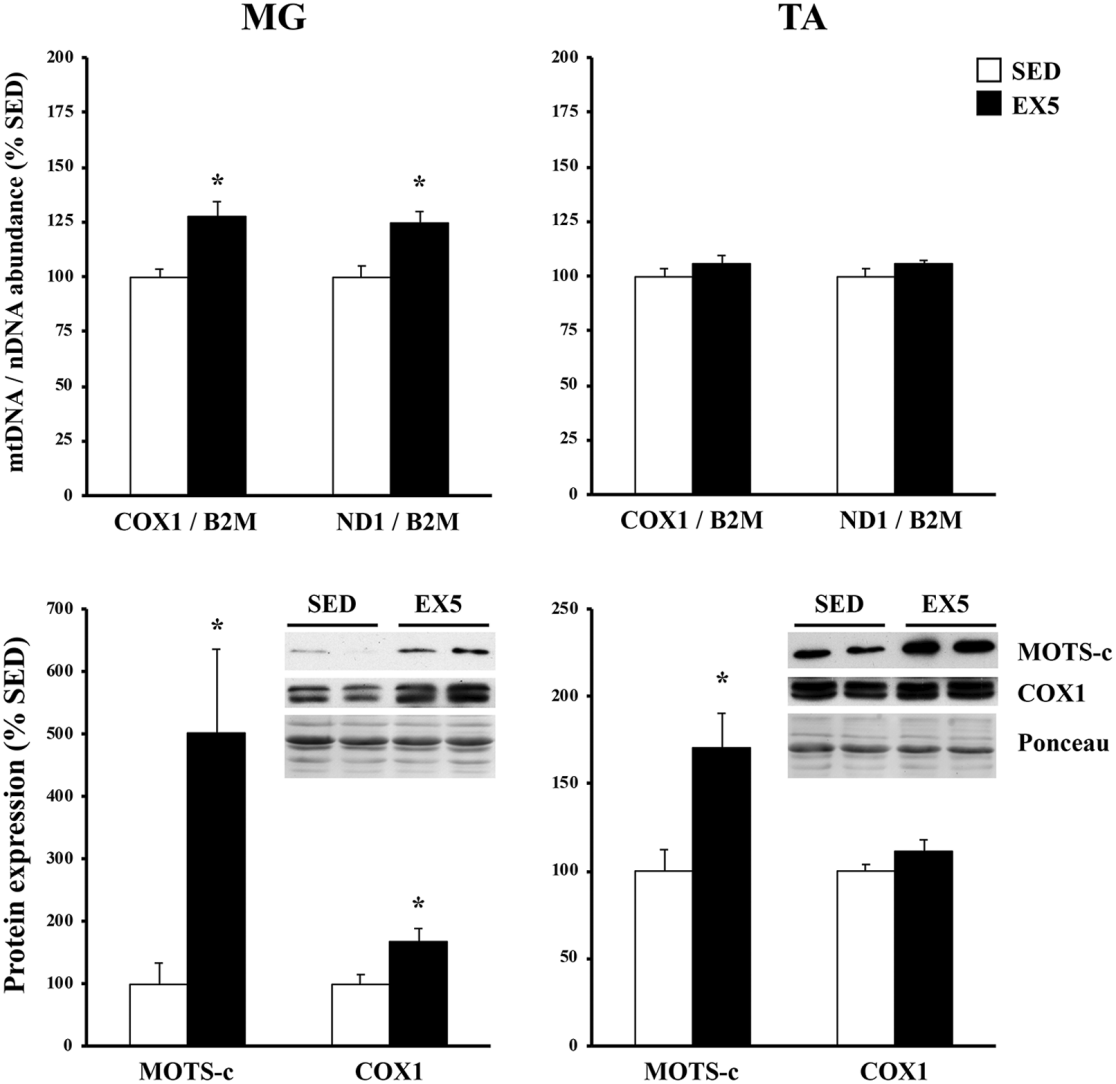
Muscle‐specific changes in mitochondrial DNA (mtDNA) abundance (top) and mitochondrial protein expression (bottom) in adult female C57BL/6j mouse medial gastrocnemius (MG) and tibialis anterior (TA) muscles after long‐term voluntary aerobic training. The mtDNA‐to‐nuclear DNA (nDNA) ratio was compared between sedentary (SED; *n* = 6) and 5‐week‐trained mice (EX5; *n* = 6) using mitochondrial genes cytochrome c oxidase subunit I (COX1) and ATP synthase subunit 6 (ATP6) and the nuclear housekeeping gene, β2 microglobulin (B2M). Expression of MOTS‐c and COX1 proteins also were compared between groups; representative protein and ponceau‐stained membranes as a loading control are shown. Values show mean ± S.E. *Statistically different from SED (*p* < 0.05).

The mtDNA‐to‐nDNA ratio was used as to determine the impact of chronic exercise on the expansion of the mitochondrial genome and, consequently, mitochondria‐encoded protein expression. Compared to SED rats, a significant increase in *ATP6*/*ACTB* and *ND1*/*ACTB* ratios were detected in EX4, EX8, and DETR6 plantaris muscles (*p* < 0.05) and trended higher in EX6 (*p* = 0.07) and DETR4 rats (*p* = 0.14) (Figure 3). Similarly, after 5 weeks of voluntary running, there was a significant increase from SED conditions in the *COX1*/*B2M* and *ND1*/*B2M* ratios in the mouse MG (*p* < 0.05) but in not the TA muscles (Figure 4).

MOTS‐c protein was detected in mouse, rat, monkey, and human skeletal muscle (Figure 2). The custom MOTS‐c antibody also demonstrated specificity against rat and mouse cardiac muscle at similar levels to the soleus muscle (data not shown). Monkey and human muscles exhibited either a more robust MOTS‐c expression or had a greater affinity to the custom antibody than rodent muscles: Human MOTS‐c protein was still readily observed at a loading content of 156 ng, whereas rat MOTS‐c was undetected below 5 μg (Figure 2). In addition, the MOTS‐c protein signal was more robust in the mouse soleus than in the plantaris or TA skeletal muscles; basal levels of MOTS‐s protein were barely observable in the mouse MG. The custom MOTS‐c antibody, however, did not demonstrate reactivity in muscle cross‐sections using immunohistochemical protocols (data not shown).

Following long‐term physical activity, MOTS‐c protein significantly increased from SED levels in rat plantaris muscles (*p* < 0.05; Figure 3). The highest MOTS‐c expression (>2‐fold) was detected in skeletal muscle of rats that trained the longest (EX8) with no subsequent period of detraining. MOTS‐c protein remained higher in DETR4 and DETR6 than SED rats after 4 or 6 weeks of detraining, respectively. Of the mitochondria‐encoded proteins assessed in the present study, only humanin exhibited a similar change as the MOTS‐c protein relative SED conditions: In all groups, rats showed higher humanin expression compared to SED animals (*p* < 0.05). Expression of COX1, ATP6, and CYTB proteins were group‐specific. Compared to SED rats, COX1 protein expression was elevated ~3.5‐, 3‐, and 2‐fold in EX4, EX6, and EX8 plantaris muscles (*p* < 0.05), respectively, but returned to SED levels in DETR4 and DETR6 conditions. ATP6 and CYTB proteins significantly increased from SED in EX4 rats (*p* < 0.05), whereas EX8 and DETR4 groups exhibited higher ATP6 and CYTB protein expression than SED rats, respectively (*p* < 0.05).

Because MOTS‐c and COX1 proteins showed the greatest change from SED in exercise‐trained rat muscles, subsequent analyses focused on these targets in exercise‐trained mouse MG and TA muscles (Figure 4). Compared to SED mice, MOTS‐c protein increased ~5‐ and 1.7‐fold in EX5 MG and TA muscles, respectively (*p* < 0.05). COX1 protein was significantly elevated from SED conditions in EX5 MG (1.7‐fold), but not in TA, muscles.

### 3.3 A single dose of exogenous MOTS‐c administration improves exercise performance in young adult mice

**FIGURE 5:**
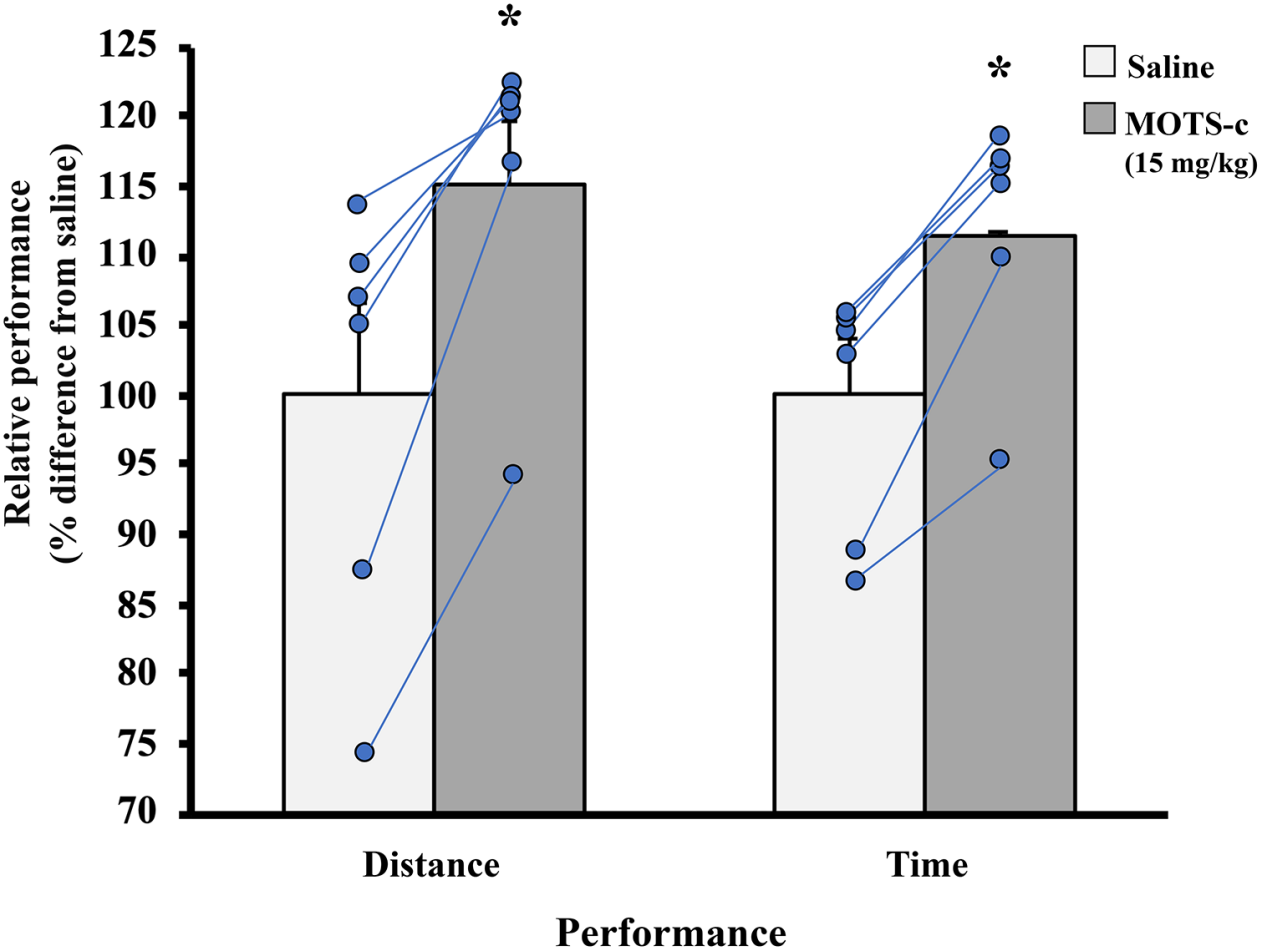
Performance enhancement in adult female C57BL/6j mice (*n* = 6) following an acute supplementation of the MOTS‐c peptide. In a cross‐over design, mice received either 0.9% saline or MOTS‐c (15 mg/kg) protein 10 min prior to the 40‐min exercise challenge at a speed of 18 m/min. Performance was assessed as total distance and time running. No mouse reached the 40‐min threshold after saline injection and 5 of 6 mice surpassed this goal after MOTS‐c administration. Only one mouse did not to reach the 40‐min goal during either condition but it exercised ~3 min longer after receiving MOTS‐c compared to its saline condition. There was a ~15% and 12% increase in exercise performance following MOTS‐c supplementation for distance and time, respectively. Values show mean ± S.E. *Statistically different from saline injected (*p* < 0.05).

This experiment was conducted to determine whether exogenous administration of MOTS‐c (15 mg/kg) would affect the exercise performance in untrained young adult mice after a single dose. Using an identical exercise test as previously reported (Reynolds et al., 2021), the performance results for total run time and distance are shown in Figure 5. No saline‐injected mouse finished the 40‐min exercise test at a speed of 18 m/min (average total exercise time: 36.1 ± 1.5 min), whereas 5 of 6 mice exceeded this 40‐min threshold and continued the test at 23 m/min until exhaustion (average total exercise time for 5 mice: 41.4 ± 0.4 min). One mouse did not reach the 40‐min threshold after receiving either saline (31.75 min) or MOTS‐c (34.85 min). In fact, all mice performed better following MOTS‐c supplementation than with saline: On average, the MOTS‐c supplementation increased performance by ~12% and 15% of total time and exercise distance, respectively, in young adult mice.

### 3.4 MOTS‐c protein localization in mouse skeletal muscle following an acute downhill running challenge

**FIGURE 6:**
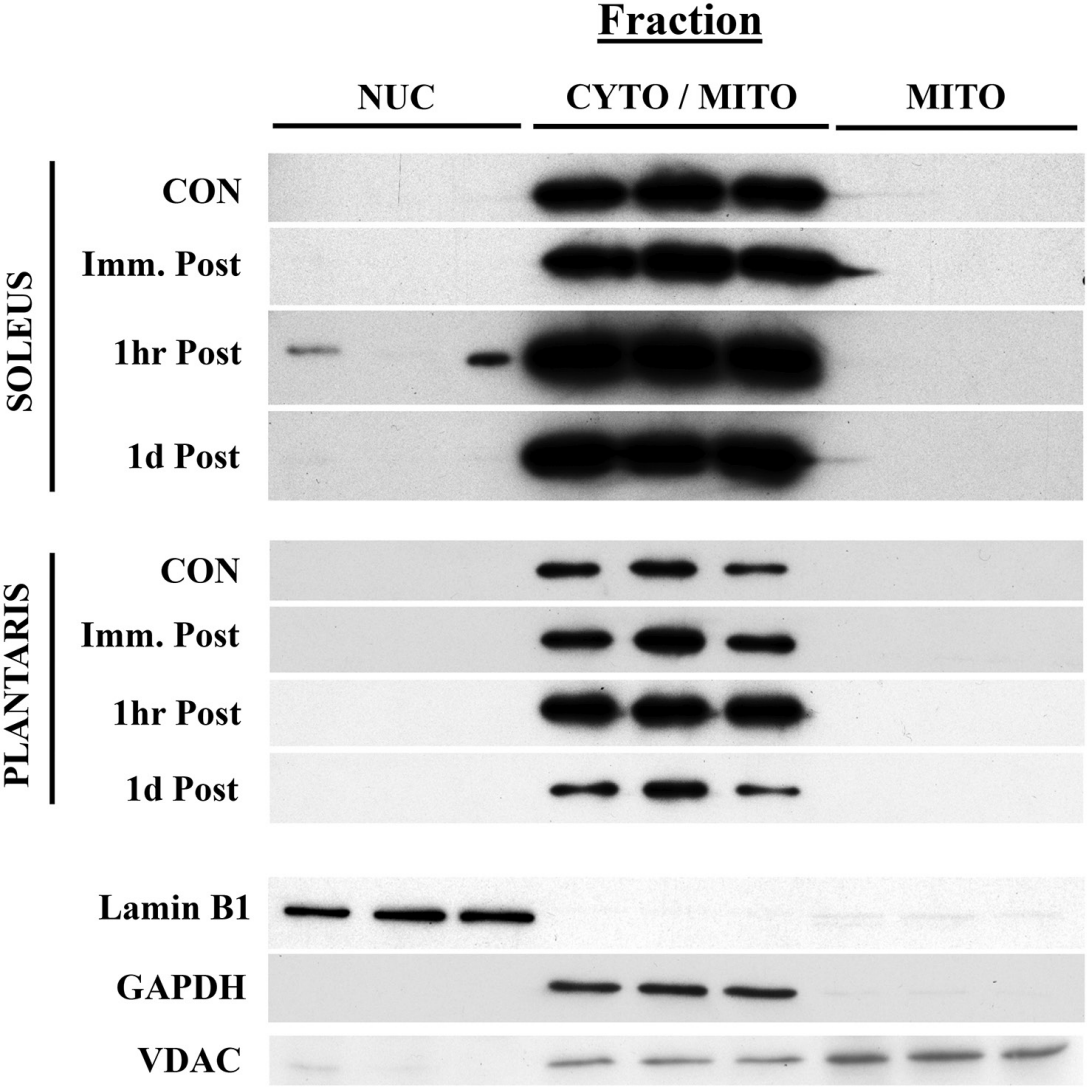
Translocation of the MOTS‐c protein following a 90‐min downhill (−15° slope) running challenge in adult female C57BL/6j mice (*n* = 6 per group). The presence of the MOTS‐c protein was qualitatively assessed in nuclear (NUC), a cytoplasmic (CYTO), and mitochondrial (MITO) fractions from fresh mouse soleus and plantaris skeletal muscles in non‐exercised control (CON) and immediately‐ (Imm.), 1 h‐, and 1d‐post downhill running. Probes against Lamin B1 (nuclear), GAPDH (cytoplasmic), and VDAC (mitochondrial) were used to confirm fractionation specificity. The cytoplasmic fraction is labeled as CYTO/MITO due to the presence of VDAC in this isolate.

The soleus and plantaris were chosen for fractionation after downhill running tests because these muscles are the highly recruited hindlimb muscles during locomotion. Initial attempts to obtain three distinct fractions from fresh muscle tissue following downhill running experiments were difficult inasmuch as the mitochondrial fraction was partially isolated with the cytoplasmic fraction due to the presence of the mitochondria‐specific marker, voltage‐dependent anion channel (VDAC) (Figure 6). Because these experiments tested whether MOTS‐c translocated to nuclei following a physiologically challenging test (Kim et al., 2018) and a distinct nuclear fraction could be obtained, as identified by presence of Lamin B1, this mixed cytoplasmic/mitochondrial fraction was considered acceptable. A distinct mitochondrial fraction, however, was still identified due to the presence of VDAC and the absence of the cytoplasmic protein, glyceraldehyde‐3‐phosphate dehydrogenase (GAPDH). In the plantaris muscles, MOTS‐c protein was detected only in the cytoplasmic/mitochondrial fraction for all post‐exercise time points (Figure 6). In the soleus muscle, MOTS‐c protein was detected in 2 of the 6 nuclear fractions at the 1 h‐post exercise (Figure 6); MOTS‐c was not detected in the nuclear fractions at any other post‐exercise time point.

## 4 DISCUSSION

MOTS‐c is a mitochondrial‐encoded peptide that improves age‐related skeletal muscle insulin resistance and enhances adaptation to metabolic stress (Kim et al., 2018, 2019; Reynolds et al., 2021). Its potential as an important regulator of systemic metabolism was highlighted in a series of studies examining changes in circulating MOTS‐c following high‐intensity exercise (Reynolds et al., 2021; von Walden et al., 2021) and by exogenously providing MOTS‐c in rodent models (Reynolds et al., 2021; Yuan et al., 2021). The goal of this study was to better understand the relationship between MOTS‐c and exercise. Here, I show that MOTS‐c protein increased within rodent skeletal muscles following long‐term voluntary physical activity and remained elevated up to 6 weeks into a detraining period. Additionally, acute exercise performance improved 12%–15% after a single exogenous dose of MOTS‐c. Taken together, these findings suggest that skeletal muscle is an important reservoir for MOTS‐c that can expand with chronic physical activity and, presumably, impact performance through its systemic release during acute exercise (Reynolds et al., 2021; von Walden et al., 2021).

In concert with D'Souza et al. (2020), MOTS‐c protein expression is highest in phenotypically slow muscle (Figure 2), which is likely related to a larger mitochondrial volume, and presumably greater mtDNA abundance, in slow than in mixed or fast muscle phenotypes (Kayar et al., 1988; Kirkwood et al., 1986, 1987; Philippi & Sillau, 1994). The mouse MG is comprised of ~80% type IIb myosin heavy chain (Talmadge et al., 2004) and contains low endogenous MOTS‐c, whereas the TA is only ~55%–60% type IIb (Agbulut et al., 2004; Allen et al., 2001) and has a higher MOTS‐c expression baseline (Figures 2 and 4). The fast‐to‐slow phenotypic shift that occurs in exercise‐trained skeletal muscles (Allen et al., 2001; Demirel et al., 1999; Fuller et al., 2006; Hyatt et al., 2019) coincides with the upregulation in MOTS‐c protein in rat plantaris and mouse MG muscles between 4 and 8 weeks of high‐volume voluntary running (Table 2) and parallels the detected rise in mtDNA abundance (Figures 3 and 4). The high levels of MOTS‐c protein detected at 4 and 6 weeks of detraining indicates that either there is a sustained expression of MOTS‐c by the expanded mitochondria or that MOTS‐c protein accumulates within the cell and is slowly turned over. The elevated MOTS‐c protein within detrained skeletal muscle reflects a lasting influence of exercise training and a potential benefit in helping to modulate glucose metabolism/insulin sensitivity (Lee et al., 2015) during the period of inactivity that follows exercise training, although there are a number of exercise‐induced adaptations that are also sustained for some duration after training stops including, but not limited to, VO_2_max, capillary density, oxidative enzymes, and cardiovascular changes (Coyle et al., 1984). The time course of physiological decompensation that occurs with detraining in relation to MOTS‐c protein, however, requires more investigation. Finally, despite an increase in MOTS‐c within the mouse TA after 5 weeks of aerobic training, there was no change in mtDNA abundance or COX1 protein expression which may reflect differences in the relative recruitment and/or adaptation of hindlimb ankle extensors (i.e., plantaris and MG muscles) versus flexors (e.g., TA muscle) to training.

Interestingly, the increase in MOTS‐c following long‐term voluntary training was not consistently mirrored by other mitochondrial‐derived (mRNA‐encoded) proteins associated with oxidative phosphorylation (OXPHOS) complexes. COX1 protein expression exhibited the greatest response to chronic exercise in rat plantaris muscle compared to relatively modest rise in ATP6 and CYTB proteins (Figure 4). Humanin, a peptide also encoded from mitochondrial rRNA, increased in trained and detrained rat plantaris muscles in a group‐specific pattern similar to that observed with MOTS‐c. There may be a couple reasons for the expression differences observed between OXPHOS‐related proteins and MOTS‐c/humanin in trained muscles. It is likely that mitochondrial mRNA‐ and rRNA‐encoded products are differentially expressed (Gustafsson et al., 2016). In fact, in vivo basal/steady‐state expression of mitochondrial rRNAs are ~50‐fold greater than mRNAs due to the relative position of two transcription promoter sites (Terzioglu et al., 2013). As the mtDNA pool expands with chronic training, the rRNA‐to‐mRNA expression differences may become more variable and distinguishable. Another possibility is that because the proteins of OXPHOS complexes are membrane‐bound and subjected to fluctuations in mitochondrial growth, expansion, damage, and degradation (Memme et al., 2019), there could be greater variability of their presence following exercise and/or detraining conditions. Conversely, MOTS‐c and humanin are not bound to membranes and may accumulate endogenously within trained and detrained muscles; this idea is supported, in part, by the age‐dependent increase MOTS‐c protein within aged human skeletal muscle (D'Souza et al., 2020). Although humanin localization was not determined in the present study, MOTS‐c protein was consistently absent in the mitochondrial fraction (Figure 6), suggesting that MOTS‐c is not limited by the boundaries of the mitochondria to accumulate within the muscle fiber. Indeed, if this accumulation hypothesis is correct, and both MOTS‐c and humanin proteins are not subjected to turnover as quickly as, for example, OXPHOS proteins, then the systemic influence on metabolism by MOTS‐c and humanin would theoretically persist during periods of physical inactivity (e.g., detraining), although it appears that MOTS‐c release from skeletal muscle is, at least, exercise‐dependent (Reynolds et al., 2021; von Walden et al., 2021). The mechanism of MOTS‐c release into the circulation from skeletal muscle, however, is currently unknown.

Improving acute exercise performance using exogenous MOTS‐c supplementation has been previously shown in young, middle‐aged, and old mice (Reynolds et al., 2021). The results presented here (Figure 5) agree with this effect with two important variations. Firstly, Reynolds et al. (2021) pre‐conditioned young mice with daily MOTS‐c injections (15 mg/kg) for 2 weeks prior to the exercise challenge. I show that the exercise performance of similarly aged mice (Reynolds et al., 2021) was enhanced using a single dose of MOTS‐c ~ 10 min prior to the start of the exercise test. Secondly, by using the same mice for saline‐ and MOTS‐c‐supplemented exercise tests in a cross‐over design, the possibility that one group of mice could be inherently outperforming another group (e.g., runners vs non‐runners) despite MOTS‐c administration is eliminated. Furthermore, the pre‐conditioning effect shown by Reynolds et al. (2021) also lends support to the notion that MOTS‐c accumulates, or is actively stored, by skeletal muscle and/or other tissues, which can then be released during an acute exercise event. A single dose injection of MOTS‐c highlights its immediate benefits on physical performance. Although it is possible that MOTS‐c enhances glucose effectiveness within skeletal muscle (Ahrén & Pacini, 2021), more work is required to determine the specific mechanism how MOTS‐c impacts performance.

In an elegant series of cell culturing experiments, Kim et al. (2018) demonstrated MOTS‐c translocation from the mitochondria into the nucleus of human kidney cells undergoing glucose restriction, serum deprivation, or conditions of oxidative stress. Once in the nucleus, MOTS‐c influences the expression of antioxidant response element‐related genes (ARE)‐related genes (Kim et al., 2018). Here, translocation of MOTS‐c into the nucleus was not commonplace in the soleus and plantaris muscles of untrained mice following a 90‐min downhill running challenge: MOTS‐c protein was detected in the nuclear fractions of only two soleus muscles (observed at the 1 h‐post exercise time point) and entirely absent in the nuclei of plantaris muscles (Figure 6). These findings suggest that while translocation can indeed take place in vivo, there are several possibilities as to why translocation was not more prevalent using this protocol. One possibility is that downhill running does not meet the threshold as “metabolically stressful” required for MOTS‐c nuclear translocation (Kim et al., 2018). Here, downhill running was used to challenge the skeletal muscle both metabolically (90 min of continuous exercise) and mechanically (−15° slope). More work is needed to investigate MOTS‐c translocation in vivo, perhaps using uphill running or high intensity interval training, which are more metabolically demanding than downhill running, is necessary (Pyne et al., 1997; Theofilidis et al., 2021). Another possibility is that the MOTS‐c protein requires a chaperone to enter the nucleus and is not detected at the expected location following western analyses (~14 kDa). Finally, it is possible that the observations made in vitro (Kim et al., 2018) do not entirely conform to those in vivo: D'Souza et al. (2020), for example, demonstrated a weak relationship between MOTS‐c protein levels and ARE‐related genes in vastus lateralis muscles of young, middle‐aged, and old human subjects. This experiment also was limited by the fact that the custom MOTS‐c antibody used here has, to date, not shown reactivity to within skeletal muscle cross sections, which could help to confirm nuclear translocation. Taken together, although nuclear translocation of the MOTS‐c protein can occur during an acute downhill exercise challenge more work is required to elucidate the physiological drivers for this event.

### 4.1 Perspective

The potential benefits of MOTS‐c on skeletal muscle and whole‐body physiology are emerging and exciting. The aim of the experiments presented here was to understand the interrelationship between skeletal muscle expression of MOTS‐c and exercise. MOTS‐c expression increases following 4–8 weeks of chronic training and remains elevated up to 6 weeks into a detraining period indicating that there is either a sustained after training has ceased or that protein accumulated in the cytoplasm and is slowly turned over. In addition, an acute exercise bout can trigger MOTS‐c to translocate into nuclear regions of skeletal muscle, although more work is needed to validate this response. Finally, MOTS‐c supplementation augments acute exercise performance ~12%–15%, suggesting that elevated MOTS‐c expression garnered with chronic training has a practical impacts for delaying the onset of fatigue/exhaustion, which would be particularly applicable in aged and/or pathological scenarios (D'Souza et al., 2020; Lee et al., 2015; Reynolds et al., 2021; Yuan et al., 2021). Additional work, however, is required to elucidate how MOTS‐c is released from skeletal muscle and improves performance and caution is warranted on the possible use of MOTS‐c as performance‐enhancing tool. Recent efforts (Knoop et al., 2019) are promising for the detection of circulating MOTS‐c in the event that this peptide is employed as a doping agent.

## ETHICS STATEMENT

All procedures and treatment protocols for animals used onsite were approved by the Arizona State University Institutional Animal Care and Use Committee in accordance with the guidelines of the American Physiological Society.

## References

[phy215377-bib-0001] Agbulut, O. , Noirez, P. , Butler‐Browne, G. , & Jockusch, H. (2004). Specific isomyosin proportions in hyperexcitable and physiologically denervated mouse muscle. FEBS Letters, 561(1–3), 191–194. 10.1016/S0014-5793(04)00179-6 15013776

[phy215377-bib-0002] Ahrén, B. , & Pacini, G. (2021). Glucose effectiveness: Lessons from studies on insulin‐independent glucose clearance in mice. Journal of Diabetes Investigation, 12(5), 675–685. 10.1111/jdi.13446 33098240 PMC8088998

[phy215377-bib-0003] Allen, D. L. , Harrison, B. C. , Maass, A. , Bell, M. L. , Byrnes, W. C. , & Leinwand, L. A. (2001). Cardiac and skeletal muscle adaptations to voluntary wheel running in the mouse. Journal of Applied Physiology, 90(5), 1900–1908. 10.1152/jappl.2001.90.5.1900 11299284

[phy215377-bib-0004] Constantin‐Teodosiu, D. , Constantin, D. , Pelsers, M. M. , Verdijk, L. B. , van Loon, L. , & Greenhaff, P. L. (2020). Mitochondrial DNA copy number associates with insulin sensitivity and aerobic capacity, and differs between sedentary, overweight middle‐aged males with and without type 2 diabetes. International Journal of Obesity, 44(4), 929–936. 10.1038/s41366-019-0473-2 31641211

[phy215377-bib-0005] Coyle, E. F. , Martin, W. H., 3rd , Sinacore, D. R. , Joyner, M. J. , Hagberg, J. M. , & Holloszy, J. O. (1984). Time course of loss of adaptations after stopping prolonged intense endurance training. Journal of Applied Physiology, 57(6), 1857–1864. 10.1152/jappl.1984.57.6.1857 6511559

[phy215377-bib-0006] Crane, J. D. , Abadi, A. , Hettinga, B. P. , Ogborn, D. I. , MacNeil, L. G. , Steinberg, G. R. , & Tarnopolsky, M. A. (2013). Elevated mitochondrial oxidative stress impairs metabolic adaptations to exercise in skeletal muscle. PLoS One, 8(12), e81879. 10.1371/journal.pone.0081879 24324727 PMC3855701

[phy215377-bib-0007] Demirel, H. A. , Powers, S. K. , Naito, H. , Hughes, M. , & Coombes, J. S. (1999). Exercise‐induced alterations in skeletal muscle myosin heavy chain phenotype: dose‐response relationship. Journal of Applied Physiology, 86(3), 1002–1008. 10.1152/jappl.1999.86.3.1002 10066716

[phy215377-bib-0008] D'Souza, R. F. , Woodhead, J. S. T. , Hedges, C. P. , Zeng, N. , Wan, J. , Kumagai, H. , Lee, C. , Cohen, P. , Cameron‐Smith, D. , Mitchell, C. J. , & Merry, T. L. (2020). Increased expression of the mitochondrial derived peptide, MOTS‐c, in skeletal muscle of healthy aging men is associated with myofiber composition. Aging, 12(6), 244–5258. 10.18632/aging.102944 PMC7138593 32182209

[phy215377-bib-0009] Ferry, A. , Amiridis, I. , & Rieu, M. (1992). Glycogen depletion and resynthesis in the rat after downhill running. European Journal of Applied Physiology and Occupational Physiology, 64(1), 32–35. 10.1007/BF00376436 1735408

[phy215377-bib-0010] Frezza, C. , Cipolat, S. , & Scorrano, L. (2007). Organelle isolation: functional mitochondria from mouse liver, muscle and cultured fibroblasts. Nature Protocols, 2(2), 287–295. 10.1038/nprot.2006.478 17406588

[phy215377-bib-0011] Fuller, P. M. , Baldwin, K. M. , & Fuller, C. A. (2006). Parallel and divergent adaptations of rat soleus and plantaris to chronic exercise and hypergravity. American Journal of Physiology. Regulatory, Integrative and Comparative Physiology, 290(2), R442–R448. 10.1152/ajpregu.00578.2005 16179485

[phy215377-bib-0012] Gustafsson, C. M. , Falkenberg, M. , & Larsson, N. G. (2016). Maintenance and expression of mammalian mitochondrial DNA. Annual Review of Biochemistry, 85, 133–160. 10.1146/annurev-biochem-060815-014402 27023847

[phy215377-bib-0013] Hesselink, M. K. , Kuipers, H. , Keizer, H. A. , Drost, M. R. , & van der Vusse, G. J. (1998). Acute and sustained effects of isometric and lengthening muscle contractions on high‐energy phosphates and glycogen metabolism in rat tibialis anterior muscle. Journal of Muscle Research and Cell Motility, 19(4), 373–380. 10.1023/a:1005345603882 9635280

[phy215377-bib-0014] Hody, S. , Warren, B. E. , Votion, D. M. , Rogister, B. , & Lemieux, H. (2022). Eccentric exercise causes specific adjustment in pyruvate oxidation by mitochondria. Medicine & Science in Sports & Exercise, in press. 10.1249/MSS.0000000000002920 35320143

[phy215377-bib-0015] Holloszy, J. O. , & Coyle, E. F. (1984). Adaptations of skeletal muscle to endurance exercise and their metabolic consequences. Journal of Applied Physiology: Respiratory, Environmental and Exercise Physiology, 56(4), 831–838. 10.1152/jappl.1984.56.4.831 6373687

[phy215377-bib-0016] Hyatt, J. P. K. , Brown, E. A. , Deacon, H. M. , & McCall, G. E. (2019). Muscle‐specific sensitivity to voluntary physical activity and detraining. Frontiers in Physiology, 10, 1328. 10.3389/fphys.2019.01328 31708796 PMC6819312

[phy215377-bib-0017] Hyatt, J. P. K. , Mattison, J. A. , & de Cabo, R. (2022). Resveratrol blunts losses in mitochondrial content in slow and to a lesser degree, mixed skeletal muscle phenotypes of non‐human primates following a long‐term high fat/sugar diet. Journal of Dietary Supplements, in press. 10.1080/19390211.2022.2039340 PMC10044467 35229700

[phy215377-bib-0018] Kayar, S. R. , Hoppeler, H. , Mermod, L. , & Weibel, E. R. (1988). Mitochondrial size and shape in equine skeletal muscle: a three‐dimensional reconstruction study. The Anatomical Record, 222(4), 333–339. 10.1002/ar.1092220405 3228204

[phy215377-bib-0019] Kim, K. H. , Son, J. M. , Benayoun, B. A. , & Lee, C. (2018). The mitochondrial‐encoded peptide MOTS‐c translocates to the nucleus to regulate nuclear gene expression in response to metabolic stress. Cell Metabolism, 28(3), 516–524.e7. 10.1016/j.cmet.2018.06.008 29983246 PMC6185997

[phy215377-bib-0020] Kim, S. J. , Miller, B. , Mehta, H. H. , Xiao, J. , Wan, J. , Arpawong, T. E. , Yen, K. , & Cohen, P. (2019). The mitochondrial‐derived peptide MOTS‐c is a regulator of plasma metabolites and enhances insulin sensitivity. Physiological Reports, 7(13), e14171. 10.14814/phy2.14171 31293078 PMC6640593

[phy215377-bib-0021] Kim, S. J. , Xiao, J. , Wan, J. , Cohen, P. , & Yen, K. (2017). Mitochondrially derived peptides as novel regulators of metabolism. The Journal of Physiology, 595(21), 6613–6621. 10.1113/JP274472 28574175 PMC5663826

[phy215377-bib-0022] Kirkwood, S. P. , Munn, E. A. , & Brooks, G. A. (1986). Mitochondrial reticulum in limb skeletal muscle. The American Journal of Physiology, 251(3 Pt 1), C395–C402. 10.1152/ajpcell.1986.251.3.C395 3752235

[phy215377-bib-0023] Kirkwood, S. P. , Packer, L. , & Brooks, G. A. (1987). Effects of endurance training on a mitochondrial reticulum in limb skeletal muscle. Archives of Biochemistry and Biophysics, 255(1), 80–88. 10.1016/0003-9861(87)90296-7 3592671

[phy215377-bib-0024] Klausen, K. , Andersen, L. B. , & Pelle, I. (1981). Adaptive changes in work capacity, skeletal muscle capillarization and enzyme levels during training and detraining. Acta Physiologica Scandinavica, 113(1), 9–16. 10.1111/j.1748-1716.1981.tb06854.x 7315443

[phy215377-bib-0025] Knoop, A. , Thomas, A. , & Thevis, M. (2019). Development of a mass spectrometry based detection method for the mitochondrion‐derived peptide MOTS‐c in plasma samples for doping control purposes. Rapid Communications in Mass Spectrometry, 33(4), 371–380. 10.1002/rcm.8337 30394592

[phy215377-bib-0050] Lee, C. , Kim, K. H. , & Cohen, P. (2016). MOTS‐c: A novel mitochondrial‐derived peptide regulating muscle and fat metabolism. Free Radical Biology and Medicine, 100, 182–187. 10.1016/j.freeradbiomed.2016.05.015 27216708 PMC5116416

[phy215377-bib-0027] Lee, H. , Kim, K. , Kim, B. , Shin, J. , Rajan, S. , Wu, J. , Chen, X. , Brown, M. D. , Lee, S. , & Park, J. Y. (2018). A cellular mechanism of muscle memory facilitates mitochondrial remodelling following resistance training. The Journal of Physiology, 596(18), 4413–4426. 10.1113/JP275308 30099751 PMC6138296

[phy215377-bib-0026] Lee, C. , Zeng, J. , Drew, B. G. , Sallam, T. , Martin‐Montalvo, A. , Wan, J. , Kim, S. J. , Mehta, H. , Hevener, A. L. , de Cabo, R. , & Cohen, P. (2015). The mitochondrial‐derived peptide MOTS‐c promotes metabolic homeostasis and reduces obesity and insulin resistance. Cell Metabolism, 21(3), 443–454. 10.1016/j.cmet.2015.02.009 25738459 PMC4350682

[phy215377-bib-0028] Li, S. , & Laher, I. (2015). Exercise pills: At the starting line. Trends in Pharmacological Sciences, 36(12), 906–917. 10.1016/j.tips.2015.08.014 26439443

[phy215377-bib-0029] Lu, H. , Tang, S. , Xue, C. , Liu, Y. , Wang, J. , Zhang, W. , Luo, W. , & Chen, J. (2019). Mitochondrial‐derived peptide MOTS‐c increases adipose thermogenic activation to promote cold adaptation. International Journal of Molecular Sciences, 20(10), 2456. 10.3390/ijms20102456 PMC6567243 31109005

[phy215377-bib-0030] Lu, H. , Wei, M. , Zhai, Y. , Li, Q. , Ye, Z. , Wang, L. , Luo, W. , Chen, J. , & Lu, Z. (2019). MOTS‐c peptide regulates adipose homeostasis to prevent ovariectomy‐induced metabolic dysfunction. Journal of Molecular Medicine, 97(4), 473–485. 10.1007/s00109-018-01738-w 30725119

[phy215377-bib-0031] Luis Araujo Minari, A. , Avila, F. , Missae Oyama, L. , & Vagner Thomatieli Dos Santos, R. (2022). Inflammatory response of the peripheral neuroendocrine system following downhill running. Cytokine, 149, 155746. 10.1016/j.cyto.2021.155746 34678553

[phy215377-bib-0032] Magalhães, J. , Fraga, M. , Lumini‐Oliveira, J. , Gonçalves, I. , Costa, M. , Ferreira, R. , Oliveira, P. J. , & Ascensão, A. (2013). Eccentric exercise transiently affects mice skeletal muscle mitochondrial function. Applied Physiology, Nutrition, and Metabolism, 38(4), 401–409. 10.1139/apnm-2012-0226 23713533

[phy215377-bib-0033] Manzanares, G. , Brito‐da‐Silva, G. , & Gandra, P. G. (2018). Voluntary wheel running: patterns and physiological effects in mice. Brazilian Journal of Medical and Biological Research, 52(1), e7830. 10.1590/1414-431X20187830 30539969 PMC6301263

[phy215377-bib-0034] Meinild Lundby, A.‐K. , Jacobs, R. A. , Gehrig, S. , de Leur, J. , Hauser, M. , Bonne, T. C. , Flück, D. , Dandanell, S. , Kirk, N. , Kaech, A. , Ziegler, U. , Larsen, S. , & Lundby, C. (2018). Exercise training increases skeletal muscle mitochondrial volume density by enlargement of existing mitochondria and not *de novo* biogenesis. Acta Physiologica, 222, e12905. 10.1111/apha.12905 28580772

[phy215377-bib-0035] Memme, J. M. , Erlich, A. T. , Phukan, G. , & Hood, D. A. (2019). Exercise and mitochondrial health. The Journal of Physiology, 599(3), 803–817. 10.1113/JP278853 31674658

[phy215377-bib-0036] Moreillon, M. , Conde Alonso, S. , Broskey, N. T. , Greggio, C. , Besson, C. , Rousson, V. , & Amati, F. (2019). Hybrid fiber alterations in exercising seniors suggest contribution to fast‐to‐slow muscle fiber shift. Journal of Cachexia, Sarcopenia and Muscle, 10(3), 687–695. 10.1002/jcsm.12410 30907516 PMC6596392

[phy215377-bib-0037] Philippi, M. , & Sillau, A. H. (1994). Oxidative capacity distribution in skeletal muscle fibers of the rat. The Journal of Experimental Biology, 189, 1–11. 10.1242/jeb.189.1.1 7964383

[phy215377-bib-0038] Poole, D. C. , Copp, S. W. , Colburn, T. D. , Craig, J. C. , Allen, D. L. , Sturek, M. , O'Leary, D. S. , Zucker, I. H. , & Musch, T. I. (2020). Guidelines for animal exercise and training protocols for cardiovascular studies. American Journal of Physiology. Heart and Circulatory Physiology, 318(5), H1100–H1138. 10.1152/ajpheart.00697.2019 32196357 PMC7254566

[phy215377-bib-0039] Pyne, D. B. , Baker, M. S. , Telford, R. D. , & Weidermann, M. J. (1997). A treadmill protocol to investigate independently the metabolic and mechanical stress of exercise. Australian Journal of Science and Medicine in Sport, 29(3), 77–82. 9302491

[phy215377-bib-0040] Rasband, W. S. (1997–2018). ImageJ. U. S. National Institutes of Health. Retrieved from https://imagej.nih.gov/ij/

[phy215377-bib-0041] Reynolds, J. C. , Lai, R. W. , Woodhead, J. S. T. , Joly, J. H. , Mitchell, C. J. , Cameron‐Smith, D. , Lu, R. , Cohen, P. , Graham, N. A. , Benayoun, B. A. , Merry, T. L. , & Lee, C. (2021). MOTS‐c is an exercise‐induced mitochondrial‐encoded regulator of age‐dependent physical decline and muscle homeostasis. Nature Communications, 12(1), 470. 10.1038/s41467-020-20790-0 PMC7817689 33473109

[phy215377-bib-0042] Short, K. R. , Vittone, J. L. , Bigelow, M. L. , Proctor, D. N. , Coenen‐Schimke, J. M. , Rys, P. , & Nair, K. S. (2005). Changes in myosin heavy chain mRNA and protein expression in human skeletal muscle with age and endurance exercise training. Journal of Applied Physiology, 99(1), 95–102. 10.1152/japplphysiol.00129.2005 15746299

[phy215377-bib-0043] Strauss, W. M. (1998). In F. M. Ausubel (Ed.), Current Protocols in Molecular Biology (pp. 2.2.1–2.2.3). Wiley.

[phy215377-bib-0044] Tajsharghi, H. , Sunnerhagen, K. S. , Darin, N. , Kyllerman, M. , & Oldfors, A. (2004). Induced shift in myosin heavy chain expression in myosin myopathy by endurance training. Journal of Neurology, 251(2), 179–183. 10.1007/s00415-004-0295-5 14991352

[phy215377-bib-0045] Talmadge, R. J. , Otis, J. S. , Rittler, M. R. , Garcia, N. D. , Spencer, S. R. , Lees, S. J. , & Naya, F. J. (2004). Calcineurin activation influences muscle phenotype in a muscle‐specific fashion. BMC Cell Biology, 5, 28. 10.1186/1471-2121-5-28 15282035 PMC509416

[phy215377-bib-0046] Terzioglu, M. , Ruzzenente, B. , Harmel, J. , Mourier, A. , Jemt, E. , López, M. D. , Kukat, C. , Stewart, J. B. , Wibom, R. , Meharg, C. , Habermann, B. , Falkenberg, M. , Gustafsson, C. M. , Park, C. B. , & Larsson, N. G. (2013). MTERF1 binds mtDNA to prevent transcriptional interference at the light‐strand promoter but is dispensable for rRNA gene transcription regulation. Cell Metabolism, 17(4), 618–626. 10.1016/j.cmet.2013.03.006 23562081

[phy215377-bib-0047] Theofilidis, G. , Bogdanis, G. C. , Stavropoulos‐Kalinoglou, A. , Krase, A. A. , Tsatalas, T. , Shum, G. , Sakkas, G. K. , Koutedakis, Y. , & Karatzaferi, C. (2021). The effects of training with high‐speed interval running on muscle performance are modulated by slope. Physiological Reports, 9(1), e14656. 10.14814/phy2.14656 33400851 PMC7785049

[phy215377-bib-0048] von Walden, F. , Fernandez‐Gonzalo, R. , Norrbom, J. , Emanuelsson, E. B. , Figueiredo, V. C. , Gidlund, E. K. , Norrbrand, L. , Liu, C. , Sandström, P. , Hansson, B. , Wan, J. , Cohen, P. , & Alkner, B. (2021). Acute endurance exercise stimulates circulating levels of mitochondrial‐derived peptides in humans. Journal of Applied Physiology, 131(3), 1035–1042. 10.1152/japplphysiol.00706.2019 34351816 PMC12854548

[phy215377-bib-0049] Yuan, J. , Wang, M. , Pan, Y. , Liang, M. , Fu, Y. , Duan, Y. , Tang, M. , Laher, I. , & Li, S. (2021). The mitochondrial signaling peptide MOTS‐c improves myocardial performance during exercise training in rats. Scientific Reports, 11(1), 20077. 10.1038/s41598-021-99568-3 34635713 PMC8505603

